# Low-Dose Radiation Yields Lower Rates of Pathologic Response in Esophageal Cancer Patients

**DOI:** 10.1245/s10434-023-14810-8

**Published:** 2024-01-10

**Authors:** Styliani Mantziari, Hugo Teixeira Farinha, Marguerite Messier, Michael Winiker, Pierre Allemann, Esat Mahmut Ozsahin, Nicolas Demartines, Guillaume Piessen, Markus Schäfer

**Affiliations:** 1 Department of Visceral Surgery, Lausanne University Hospital CHUV, Lausanne, Switzerland; 2 Faculty of Biology and Medicine, University of Lausanne UNIL, Lausanne, Switzerland; 3 Department of Digestive and Oncological Surgery, CHU Lille, Lille, France; 4 Department of Radiation Oncology, Lausanne University Hospital CHUV, Lausanne, Switzerland; 5 CNRS, Inserm, UMR9020-U1277 - CANTHER – Cancer Heterogeneity Plasticity and Resistance to Therapies, CHU Lille, Univ. Lille, Lille, France

**Keywords:** Esophageal cancer, Neoadjuvant treatment, Rradiotherapy, Pathologic response, Survival

## Abstract

**Background:**

Although neoadjuvant chemoradiation (nCRT) followed by surgery is standard treatment for locally advanced esophageal or gastroesophageal junction (E/GEJ) cancer, the optimal radiation dose is still under debate.

**Objective:**

The aim of this study was to assess the impact of different preoperative radiation doses (41.4 Gy, 45 Gy or 50.4 Gy) on pathologic response and survival in E/GEJ cancer patients.

**Methods:**

All consecutive patients with E/GEJ tumors, treated with curative intent between January 2009 and December 2016 in two referral centers were divided into three groups (41.4 Gy, 45 Gy and 50.4 Gy) according to the dose of preoperative radiotherapy. Pathologic complete response (pCR) rates, postoperative morbidity, overall survival (OS) and disease-free survival (DFS) were compared among the three groups, with separate analyses for adenocarcinoma (AC) and squamous cell carcinoma (SCC).

**Results:**

From the 326 patients analyzed, 48 were included in the 41.4 Gy group (14.7%), 171 in the 45 Gy group (52.5%) and 107 in the 50.4 Gy group (32.8%). Postoperative complication rates were comparable (*p* = 0.399). A pCR was observed in 15%, 30%, and 34% of patients in the 41.4 Gy, 45 Gy and 50.4 Gy groups, respectively (*p* = 0.047). A 50.4 Gy dose was independently associated with pCR (odds ratio 2.78, 95% confidence interval 1.10–7.99) in multivariate analysis. Within AC patients, pCR was observed in 6.2% of patients in the 41.4 Gy group, 29.2% of patients in the 45 Gy group, and 22.7% of patients in the 50.4 Gy group (*p* = 0.035). No OS or DFS differences were observed.

**Conclusions:**

A pCR was less common after a preoperative radiation dose of 41.4 Gy in AC patients. Radiation dose had no impact on postoperative morbidity, long-term survival, and recurrence.

**Supplementary Information:**

The online version contains supplementary material available at 10.1245/s10434-023-14810-8.

Neoadjuvant chemoradiation (nCRT) followed by surgery is widely used for locally advanced (>cT2 and/or N+) esophageal cancer in the Western world, offering prolonged long-term survival compared with upfront surgery.^1,2^ Although perioperative chemotherapy has proven its efficacy for adenocarcinoma (AC) since the FLOT trial,^3^ nCRT remains a valuable treatment option, improving pathologic complete response (pCR), R0 resection rates, and long-term survival.^4,5^ Low-dose (41.4 Gy) RT combined with carboplatin-paclitaxel-based chemotherapy has become the standard of care after publication of the CROSS trial, offering pCR in 29% of all patients, 49% in squamous cell carcinoma (SCC) patients and 23% in AC patients.^6^ However, several nCRT combinations have been previously used, with variable results. The Swedish NeoRes trial reported a pCR rate of 28% with 40 Gy and platin-5-fluorouracil chemotherapy,^4^ whereas Stahl et al. found a pCR rate of 15.6% with a 30 Gy RT dose.^5^ The Swiss SAKK 75/02 trial reported a pCR rate of 23% (38% for SCC and 16% for AC) after nCRT with docetaxel, cisplatin and 45 Gy,^7^ whereas another retrospective series showed no differences in pCR rate comparing 41.4 Gy versus 50.4 Gy for both SCC and AC.^8^ Thus, despite the proven efficacy of different doses of preoperative radiation, comparative studies are scarce and results remain contradictory. This question becomes of particular clinical importance as the watch-and-wait strategy is gaining interest as a curative treatment option in esophageal cancer.^9^ van der Wilk et al. demonstrated similar rates of locoregional and distant recurrence for patients undergoing definitive chemoradiation and surgery-on-demand, compared with nCRT and upfront surgery.^10^

Two previous studies^11,12^ assessed survival according to the preoperative RT dose (41.4 Gy, 45 Gy, or 50.4 Gy) but no significant differences were found. Although such small differences of RT doses are hardly be expected to entail a clear survival benefit, histological response represents a highly relevant outcome *per se* as it might increase the chances of a successful watch-and-wait strategy, especially for the more radio-resistant AC. The potential effect of increased radiation dose on postoperative and long-term morbidity needs to be considered as a higher dose of radiation may increase the risk of anastomotic leakage and surgical site infections.^13^ Late toxicities, such as radiation-induced pneumonitis, cardiotoxicity, and esophageal fibrosis have been reported in up to 20% of SCC patients with a 60 Gy/50.4 Gy RT dose,^14^ but this aspect remains poorly documented in patients with nCRT.

The aim of this study was to assess the impact of preoperative radiotherapy dose (41.4 Gy, 45 Gy, or 50.4 Gy) on pCR, postoperative morbidity, and long-term survival in patients with esophageal or gastroesophageal junction (E/GEJ) tumors, with a subgroup analysis according to histological type.

## Patients and methods

All consecutive patients with esophageal cancer treated with curative intent between January 2009 and December 2016 in two tertiary referral centers were retrospectively assessed. Inclusion criteria were AC and squamous cell cancer histological type, treated with neoadjuvant chemoradiotherapy (nCRT) and surgery. Patients with other histological types, emergency surgery, perioperative chemotherapy, salvage esophagectomy after definitive chemoradiotherapy, delayed surgery >15 weeks after the end of chemoradiation, and total radiation dose <41 Gy or >50.4 Gy were excluded from the study. Demographic, surgical, and oncological data were retrieved from prospectively maintained institutional databases. The primary outcome was defined as the histological response to treatment, whereas secondary outcomes were postoperative morbidity, overall survival, and DFS. The study was approved by the Ethics Committee of both participating centers (CER-VD ID v42017-02-17).

### Tumor Staging and Treatment Details

Initial diagnostic work-up was performed with oeso-gastroduodenoscopy and biopsies, endoscopic ultrasound, thoracoabdominal CT scan, and total 18F-fludeoxyglucose-positron emission tomography/computed tomography (FDG-PET/CT) scan for detection of distant metastases. The 7th UICC/TNM system^15^ was used for clinical staging. All cases were discussed in each institution’s multidisciplinary tumor board to define the treatment. Local advanced lesions (cT3 and/or N+) underwent neoadjuvant treatment followed by surgery.^2^ Restaging was performed 4 weeks after the end of treatment, with thoracoabdominal CT, FDG-PET/CT and endoscopy, and surgery was planned 4–8 weeks later. Specific assessment protocols for clinical complete response were not established as the watch-and-wait strategy was not a standard treatment option. Histologic response to treatment was assessed using the Mandard tumor regression grade (TRG) score, with TRG 1 representing pCR.^16^ Postoperative complications were graded according to the Clavien–Dindo system.^17^

Chemotherapy protocols were based on 5-fluorouracil-cisplatin and carboplatin-paclitaxel, while radiation dose ranged between 41.4 Gy and 50.4 Gy. Low-dose (41.4 Gy) radiation was introduced in the neoadjuvant context after publication of the CROSS trial in 2012.^6^ Radiation was administered to a total dose of 41.4 Gy, 45 Gy, or 50.4 Gy in fractions of 1.8 Gy. Although both participating centers shifted towards lower dose RT (41.4 Gy and 45 Gy) after 2012, universal treatment protocols were not imposed. The choice to maintain higher radiation doses in some patients was physician-dependent and not driven by a formal change of practice away from the CROSS regimen. In addition, several patients referred for surgery in the two participating centers had received nCRT in other institutions, where the CROSS regimen had not been clearly established.

At the time of this study, oncologic esophagectomy was performed by either the open or hybrid thoracoabdominal approach, as thoracoscopy was not yet introduced in current practice. The standard surgical approach was thoracoabdominal Lewis resection for middle-distal third tumors, and three-field McKeown resection for upper and middle-third lesions.

### Statistical Analysis

Data were summarized using frequencies (%) for categorical variables, and median (interquartile range [IQR]) or mean (standard deviation [SD]) for continuous variables. Survival and recurrence were expressed as the median, in postoperative months (95% confidence intervals [CIs]). Intergroup comparisons were performed using the Chi-square or Fisher’s exact test for categorical variables and analysis of variance (ANOVA) tests for continuous variables. A multivariate logistic regression model was used to define predictors of pathological complete response (pCR). Survival was assessed using the Kaplan–Meier method and the log-rank test, whereas a Cox regression model was used to identify variables independently related to overall survival (OS). Covariates with a *p*-value <0.1 on a univariate level were included in the multivariate analysis. Based on the different radio-sensibility of AC and SCC, subgroup analyses were performed according to histological type. Statistical analysis was performed using R studio version 1.1.383 (Boston, MA, USA) and SPSS version 23.0 (IBM Corporation, Armonk, NY, USA) software.

## Results

**Fig. 1:**
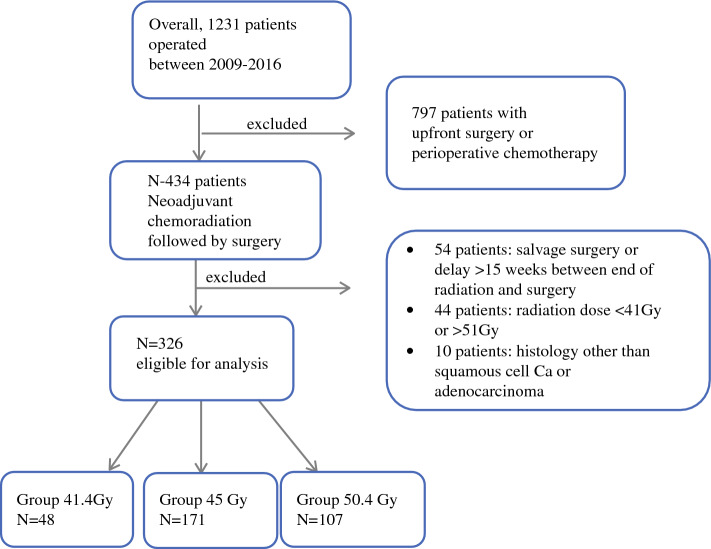
Selection of patients included in the study. *Gy* Gray, *Ca* carcinoma

Overall, 326 patients fulfilled the inclusion criteria and were eligible for the present analysis (Fig. 1). The 41.5 Gy group consisted of 48 patients (14.7%), the 45 Gy group consisted of 171 patients (52.5%), and the 50.4 Gy group consisted of 107 patients (32.8%).

Demographic characteristics for all patients are displayed in Table 1. The 41.4 Gy group included more GEJ tumors and AC histology compared with the 45 Gy and 50.4 Gy groups. Additionally, more advanced cT stages (cT3-T4) were present in the 41.4 Gy group (92%, 78%, and 79% respectively; *p* = 0.001), although nodal status (cN) was comparable (*p* = 0.407). Chemotherapy type was more often carboplatin-paclitaxel in the 41.4 Gy group (73%), and 5-fluorouracil-cisplatin in the 45 Gy and 50.4 Gy groups (47% and 44%) [*p* < 0.001].

**Table 1:** Patient demographics and baseline characteristics according to preoperative radiation dose

	All patients [*N* = 326]	41.4 Gy group [*n* = 48]	45 Gy group [*n* = 171]	50.4 Gy group [*n* = 107]	*p*-Value
Mean age, years	61 [10]	61 [11]	62 [10]	61 [10]	0.751
Male sex	258 (79.1)	35 (73)	139 (81)	84 (79)	0.443
ASA class					0.876
I–II	238 (73)	32 (66)	130 (76)	76 (71)	
III–IV	83 (25.5)	15 (31)	39 (23)	29 (27)	
Histology					
SCC	178 (54.6)	16 (33)	99 (58)	63 (59)	0.006
AC	148 (45.4)	32 (67)	72 (42)	44 (41)	
Tumor location					0.017
Upper esophagus	13 (4)	0 (0)	9 (5)	4 (4)	
Middle esophagus	113 (34.7)	14 (29)	57 (33)	42 (39)	
Lower esophagus	104 (31.9)	9 (19)	58 (34)	37 (35)	
GEJ	95 (29.1)	25 (52)	46 (27)	24 (22)	
Preoperative weight loss^a^					0.040
<10%	194 (59.5)	21 (43)	114 (66)	59 (55)	
10–20%	81 (24.8)	12 (25)	41 (24)	28 (26)	
>20%	16 (4.9)	5 (10)	5 (3)	6 (6)	
Active smoking	220 (67.5)	28 (58)	117 (68)	75 (70)	0.504
cT stage					0.001
1–2	56 (17.2)	3 (6)	36 (22)	17 (16)	
3–4	268 (82.2)	44 (92)	134 (78)	90 (85)	
cN stage					0.407
0	86 (26.4)	11 (23)	49 (28)	26 (24)	
1	229 (70.2)	34 (71)	119 (70)	76 (71)	
2–3	11 (3.4)	3 (6)	3 (2)	5 (5)	
Chemotherapy type					<0.001
5-fluorouracil-cisplatin	130 (39.9)	3 (6)	80 (47)	47 (44)	
FOLFOX	89 (27.3)	10 (21)	51 (30)	28 (26)	
Carbo-taxol	77 (23.6)	35 (73)	22 (13)	20 (19)	
Other	22 (6.7)	0	13 (8)	9 (8)	
Postoperative chemotherapy	17 (5.2)	0 (0)	14 (8)	3 (3)	0.031

Data are expressed as frequency (%) or mean [SD]

*AC* adenocarcinoma, *ASA* American Society of Anesthesiologists, *GEJ* gastroesophageal junction, *SCC* squamous cell cancer, *SD* standard deviation

^a^Percentage of weight loss on diagnosis compared with baseline weight

### Surgery and Postoperative Outcomes

A thoracoabdominal Ivor–Lewis resection was most commonly performed in all patients (*p* = 0.095). The hybrid (laparoscopic) approach was more frequently employed in the 41.4 Gy group (77% vs. 50% in the 45 Gy group and 57% in the 50.4 Gy group; *p* = 0.004). No differences were observed in terms of operative time, intraoperative blood loss or postoperative complications, particularly anastomotic leak rates and respiratory and cardiac complications. In-hospital mortality was also similar between groups (2%, 6%, and 3%, respectively; *p* = 0.352) [Table 2].

**Table 2:** Surgical characteristics and postoperative outcomes according to preoperative radiation dose

	All patients [*N* = 326]	41.4 Gy group [*n* = 48]	45 Gy group [*n* = 171]	50.4 Gy [*n* = 107]	*p*-value
Operative technique					
Thoracoabdominal Lewis	278 (85.3)	42 (88)	150 (88)	86 (80)	0.095
Three-field esophagectomy	39 (12)	3 (6)	18 (11)	18 (17)	
Total esogastrectomy	5 (1.5)	1 (2)	2 (1)	2 (2)	
Transhiatal esophagectomy	3 (0.9)	2 (4)	0 (0)	1(1)	
Laparoscopic approach	184 (56.4)	37 (77)	86 (50)	61 (57)	0.004
Operative time, min	336 [100.9]	317 [66]	334 [100]	344 [90.3]	0.251
Estimated blood loss, mL	411.8 [587]	297 [354]	425 [645]	354 [284]	0.360
Major complications^a^	87(26.7)	9 (19)	47 (27)	31 (29)	0.399
Oesobronchic fistula	7 (2.1)	2 (4)	5 (3)	0 (0)	0.152
Anastomotic leak	56 (17.2)	10 (21)	27 (16)	19 (18)	0.702
Conduit necrosis	14 (4.3)	1 (2)	8 (5)	5 (5)	0.715
Chylothorax	20 (6.1)	5 (10)	9 (5)	6 (6)	0.405
Respiratory complications	149 (45.7)	20 (42)	79 (46)	50 (47)	0.828
Pneumonia	79 (24.2)	15 (31)	33 (19)	31 (29)	0.088
Respiratory failure	44 (13.5)	8 (17)	24 (14)	12 (11)	0.762
Cardiac failure	13 (4)	1 (2)	7 (4)	5 (5)	0.828
Cardiac arrythmia	55 (16.9)	8 (17)	24 (14)	23 (21)	0.271
Venous thromboembolism	19 (5.8)	1 (2)	8 (5)	10 (9)	0.186
Length of hospital stay, days	21.9 [27]	23.2 [28.9]	20.7 [18.3]	22.9 [25.2]	0.666
In-hospital mortality	15 (4.6)	1 (2)	11 (6)	3 (3)	0.352

Data are expressed as frequency (%) or mean [standard deviation] as appropriate

^a^Defined as Clavien–Dindo grade >IIIa

### Histopathologic Results, Tumor Response to Treatment

The 41.4 Gy group included more poorly differentiated tumors (G3) compared with the other two groups, whereas pT stage was similar (Table 3). The 50.4 Gy group had higher rates of pN0 status (69% vs. 54% for the 41.4 Gy group and 58% for the 45 Gy group; *p* = 0.041). R0 resection rates were similar between groups (*p* = 0.533). Patients who received low-dose radiotherapy (41.4 Gy) had lower rates of pCR (15%, *n* = 7) compared with the 45 Gy group (30%, *n* = 52), and the 50.4 Gy group (34%, *n* = 36) [*p* = 0.047].

**Table 3:** Histopathologic results according to preoperative radiation dose

	All patients [*N* = 326]	41.4 Gy group [*n* = 48]	45 Gy group [*n* = 171]	50.4 Gy group [*n* = 107]	*p*-value
pT stage					0.141
0	99 (30.4)	7 (15)	56 (33)	36 (34)	
1	39 (12)	5 (10)	22 (13)	12 (11)	
2	46 (14.1)	6 (13)	24 (14)	16 (15)	
3	136 (41.7)	30 (63)	66 (39)	40 (37)	
4	6 (1.8)	0 (0)	3 (2)	3 (3)	
pN stage					0.041
0	199 (61)	26 (54)	99 (58)	74 (69)	
1	81 (24.8)	16 (33)	49 (29)	16 (15)	
2	34 (10.4)	6 (13)	18 (11)	10 (9)	
3	12 (3.7)	0 (0)	5 (3)	7 (7)	
Differentiation grade (G)					<0.001
1	66 (20.2)	4 (8)	41 (24)	21 (20)	
2	102 (31.3)	21 (44)	41 (24)	40 (37)	
3	64 (19.6)	13 (27)	31 (18)	20 (19)	
Harvested lymph nodes	23.4 [10]	25 [10]	23 [10]	21 [10]	0.067
Mandard TRG					
1–2	178 (54.6)	21 (44)	95 (56)	62 (58)	0.032
3–5	142 (43.6)	26 (54)	75 (44)	41 (38)	
pCR	95 (29.1)	7 (15)	52 (30)	36 (34)	0.047
Resection margins					
R0	301 (92.3)	46 (96)	158 (92)	97 (91)	0.533
R1/2	26 (7.7)	2 (4)	13 (8)	10 (9)	

Results are presented as frequency (%) or mean [standard deviation] as appropriate

*pCR* pathologic complete response, *TRG* tumor regression grade

Logistic regression was performed to identify independent predictors of pCR. Patients with 50.4 Gy had an increased chance of obtaining pCR compared with the 41.4 Gy group (adjusted odds ratio [OR] 2.78, 95% CI 1.10–7.99); among the other parameters, only cT status and cN status remained independent predictors of pCR (Table 4).

**Table 4:** Logistic regression analysis of predictors of complete pathologic response

	Unadjusted OR	95% CI	*p*-Value	Adjusted OR	95% CI	*p*-Value
cT stage						
1	1			1		
2	0.12	0.01–0.81	0.063	0.09	0.004–0.66	0.041
3–4	0.06	0.002–0.34	0.008	0.04	0.002–0.29	0.006
cN stage						
0	1			1		
1	1.86	1.04–3.47	0.042	1.73	0.94–3.33	0.086
2	5.07	1.22–22.5	0.025	7.02	1.53–35.1	0.013
3	4.09	0.15–106.2	0.331	4.28	0.16–115.4	0.322
Tumor location						
GEJ	1			1		
Distal third	2.26	1.19–4.42	0.014	1.43	0.66–3.10	0.366
Middle third	2.17	1.15–4.20	0.019	0.98	0.39–2.41	0.959
Upper third	1.28	0.27–4.72	0.745	0.48	0.08–2.27	0.379
Radiotherapy dose, Gy						
41.4	1			1		
45	2.56	1.14–6.57	0.033	2.30	0.94–6.46	0.087
50.4	2.97	1.27–7.82	0.017	2.78	1.10–7.99	0.040
Histology						
SCC	1			1		
AC	0.54	0.32–0.87	0.014	0.60	0.29–1.20	0.154

*OR* odds ratio, *GEJ* gastroesophageal junction, *Gy* Gray, *SCC* squamous cell cancer, *AC* adenocarcinoma, *CI* confidence interval

### Overall Survival and Disease-Free Survival

**Fig. 2:**
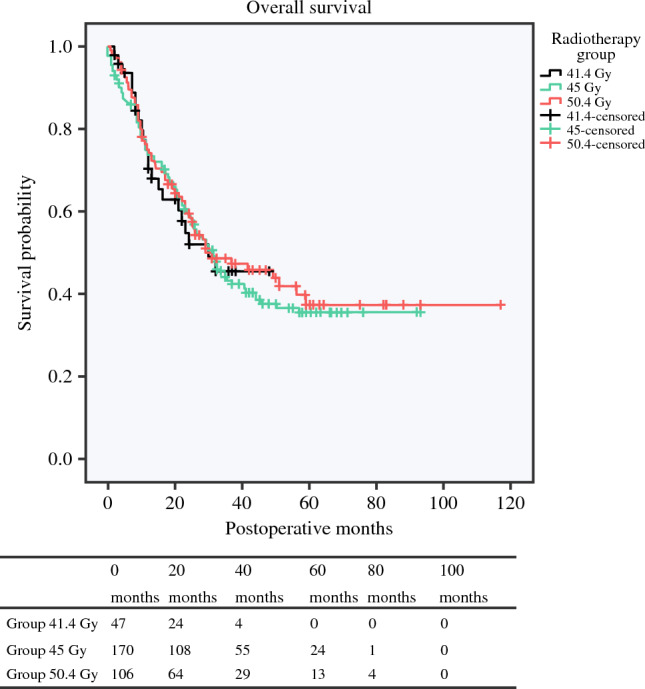
Kaplan–Meier curves of overall survival for the three radiation groups. Median overall survival was 30.0 months (95% CI 21–not available) for the 41.4 Gy group, 31.6 months (95% CI 25.5–37.5) for the 45 Gy group, and 30.0 months (95% CI 9.9–50.1) for the 50.4 Gy group (*p *= 0.902). *CI* confidence interval, *Gy* Gray

**Fig. 3:**
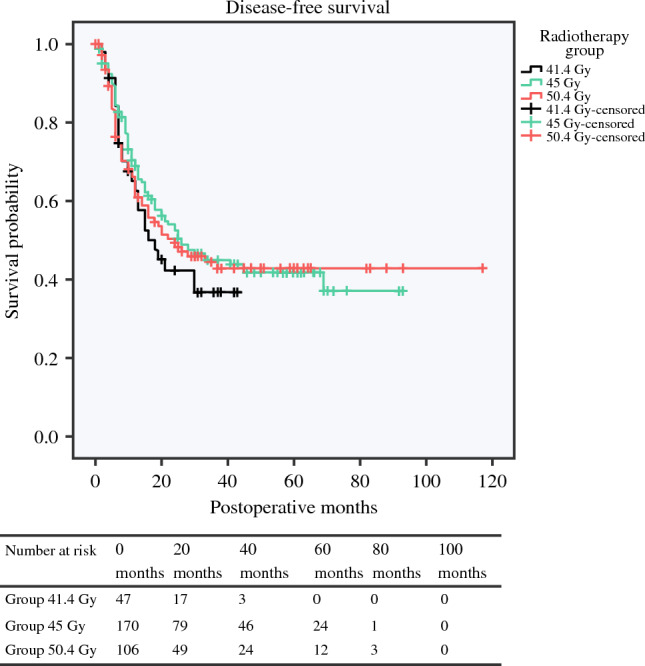
Kaplan–Meier curves of disease-free survival for the three radiation groups. Median disease-free survival was 18.0 (95%CI 9.9–26.0) months in the 41.4 Gy group, 26.0 (95% CI 15.9–36.1) months in the 45 Gy group, and 24.0 (95% CI 9.0–38.9) months in the 50.4 Gy group (*p* = 0.630). *CI* confidence interval, *Gy* Gray

OS did not show significant differences among the three groups, with a median of 30.0 months (95% CI 21–not available) for patients in the 41.4 Gy group, 31.6 months (95% CI 25.5–37.5) for patients in the 45 Gy group, and 30.0 months (95% CI 9.9–50.1) for patients in the 50.4 Gy group (Fig. 2). In multivariate Cox regression, radiotherapy dose did not remain significant; however, pN2 stage (hazard ratio [HR] 1.98, 95% CI 1.25–3.13), major (Clavien–Dindo >IIIA) postoperative complications (HR 2.18, 95% CI 1.58–3.01), and positive resection margins (HR 2.26, 95% CI 1.35–2.76) were independent predictors of poor long-term survival (electronic supplementary material 1). Median DFS was also comparable—18.0 (95% CI 9.9–26.0) months for the 41.4 Gy group, 26.0 (95% CI 15.9–36.1) months for the 45 Gy group, and 24.0 (95% CI 9.0–38.9) months for the 50.4 Gy group (*p* = 0.630) [Fig. 3].

### Subgroup Analysis by Histological Type

#### Squamous Cell Carcinoma

SCC patients presented similar rates of pCR, irrespective of the total amount of radiation received. In the 41.4 Gy group, 31.5% (*n* = 5) of patients had a pCR, versus 31.3% (*n* = 31) of patients in the 45 Gy group and 41.3% (*n* = 26) of patients in the 50.4 Gy group (*p* = 0.410). Median OS did not present significant differences (25.6 months [95% CI 17.3–34.0] in the 41.4 Gy group, 44.7 months [95% CI 37.2–52.3] in the 45 Gy group, and 55.2 months [95% CI 42.1–68.3] in the 50.4 Gy group; *p* = 0.977). DFS was also comparable (41.4 Gy group: 15.0 months [95% CI 9.7–20.3]; 45 Gy group: 24.0 months [95% CI 10.1–37.9]; 50.4 Gy group: 20 months [95% CI 8.3–31.7]; *p* = 0.807)

#### Adenocarcinoma

Total radiation dose was associated with significant differences on tumor response in the AC subgroup; 6.2% (*n* = 2) of patients in the 41.4 Gy group had a pCR, versus 29.2% (*n* = 21) in the 45 Gy group and 22.7% (*n* = 10) in the 50.4 Gy group (*p* = 0.035). Median OS was 30.0 months (95% CI 17.9–42.1) in the 41.4 Gy group, 31.4 months (95% CI 16.7–46.2) in the 45 Gy group, and 49 months (95% CI 25.8–72.2) in the 50.4 Gy group (*p* = 0.690). Respective DFS was 18 months (95% CI 4.8–31.6), 30 months (95% CI 6.5–53.5), and 33 months (95% CI 7.5–58.5) in the 41.4 Gy, 45 Gy, and 50.4 Gy groups (*p* = 0.644).

## Discussion

In the present study, pCR was more common after high-dose radiation (50.4 Gy or 45 Gy), especially in patients with AC, although this had no significant impact on long-term survival and recurrence rates. Dose escalation from 41.4 Gy to 50.4 Gy did not increase postoperative mortality or morbidity rates.

Although 50% of esophageal cancer patients present with a curable disease stage, overall reported survival remains rather poor (20% 5-year OS).^18,19^ Since the early 2000s, neoadjuvant treatment (radiochemotherapy [nCRT] or chemotherapy [nCT]) followed by esophagectomy is the standard of care for locally advanced E/GEJ cancer in most Western countries.^20^ Early studies suggested that nCRT offered a survival advantage over nCT or surgery alone, however systemic treatment has known substantial progress in the meantime.^21,22^ Preliminary results of the randomized NeoAegis trial comparing nCRT versus nCT showed better histologic response in nCRT patients, without significant survival differences.^23^ Indeed, nCRT has been previously related to better tumor regression, but survival benefit remains contradictory.^24–27^ If histologic response represents a proxy of treatment efficacy and long-term outcomes, the adjunct of radiation in preoperative chemotherapy seems a reasonable approach. In this context, a better understanding of the optimal radiation dose in an nCRT context is needed. North American protocols recommend an nCRT dose of 50.4 Gy,^28,29^ while European (European Society for Medical Oncology [ESMO]) guidelines suggest nCRT with 41.4 Gy for SCC, but do not clearly specify radiation dose in AC, although the low-dose CROSS protocol is also suggested.^2^ The present study illustrates suboptimal histological response rates with low-dose radiation in AC patients. Previously, Nabavizadeh et al. showed similar pCR after the modified CROSS regimen, using 50.4 Gy compared with the standard low dose, despite an increased risk of severe radiation-induced acute lung injury in the higher dose.^30^ In another study including 80% AC and 20% SCC, Ji et al showed that low-dose radiation (41.4 Gy) may even offer improved OS compared with higher-dose regimens, with similar local control and cT and N status downstaging.^31^ Although the reason behind the survival benefit observed in the low-dose group remains unclear, increased treatment-related mortality may be part of the explanation.

Treatment-related toxicity is an important issue to consider when defining the optimal radiation dose. In the present study, postoperative morbidity/mortality rates were not increased in the high-dose radiation group, however specific data on nCRT toxicity are not available. It has previously been reported that up to 14% of patients who started nCRT for esophageal cancer will not be able to proceed to surgery due to disease progression or reduction of physical functioning and treatment-related toxicity.^32^ In a definitive chemoradiation context, the French FREGAT group had suggested that when radiation dose escalates to 55 Gy, patients experienced increased major postoperative morbidity, anastomotic leakage rates and mortality, and even reduced OS.^33^ Stahl et al. showed equivalent survival in SCC patients treated with exclusive high-dose chemoradiation (65 Gy) alone versus low-dose nCRT followed by surgery (40 Gy), with less treatment-related mortality in the nCRT arm.^34^ The CONCORDE/PRODIGE 26 trial showed that 66 Gy, although not entailing more toxicity than 50 Gy, failed to improve progression-free survival, proposing 50 Gy as a standard dose for definitive CRT.^35^ Thus, dose escalation to >50 Gy is nowadays discouraged, whereas more recent techniques, such as the Proton Beam Therapy (PBT) have emerged to reduce off-target adverse effects by focusing dose distribution to the primary tumor.^36^

The current study has particular clinical relevance in the era of the ‘watch-and-wait’ treatment for esophageal cancer, where obtaining the best possible response after neoadjuvant treatment is key. Indeed, in patients with complete clinical response (cCR) after nCRT, delayed surgery on-demand has been suggested in case of local relapse, as it provided similar survival results to nCRT and upfront surgery.^10^ The ‘watch-and-wait’ strategy seems very promising,^9^ especially in the SCC histology, as the CROSS trial showed a pCR in almost 50% of SCC compared with 25% of AC patients.^6^ Although the challenge to reliably detect cCR after chemoradiation needs to be acknowledged,^37^ the SANO trial is currently assessing the watch-and-wait strategy in patients presenting cCR after nCRT with 41.4 Gy.^38,39^ Based on the present study’s results, the CROSS protocol radiation dose (41.4 Gy) might carry a high risk of undertreating AC lesions, as significantly lower rates of pCR were observed compared with 45–50.4 Gy.

This study has some limitations that need to be discussed. Although data are extracted from prospectively maintained databases, the retrospective character of the study entails the shortcoming of missing data, especially in terms of treatment-related toxicity. In addition, some baseline differences exist among the three radiotherapy groups, notably in the histological type, tumor location, cT stage, and chemotherapy regimen. To face this methodological drawback, we performed separate subgroup analyses by histological type for all the main outcomes (pCR, survival) due to the high clinical significance of histology. In addition, rigorous multivariable analyses adjusted for all the above-mentioned confounders when assessing the independent predictive value of radiotherapy dose on pCR and survival. Another limitation concerns the absence of clearly defined criteria for radiotherapy regimen choice. The chosen study period is anterior to recent advances in systemic treatment of esophageal cancer, such as adjuvant checkpoint inhibitors, that led to improved DFS after nCRT. Finally, radiotherapy modalities are becoming increasingly precise and efficient, and the 50.4 Gy dose previously used in the neoadjuvant setting is now outdated and only admitted as definitive CRT. However, as our data illustrate, this dose might still have its place in selected cases, as, for example, an AC patient in watch-and-wait treatment strategy.

## Conclusion

Our findings suggest that low-dose radiation is efficient in SCC; a higher dose of 45–50.4 Gy may be needed for AC as it offers increased chances for pathological complete response, without compromising postoperative outcomes. These findings are of particular clinical relevance in candidates for the watch-and-wait strategy as they suggest low-dose RT to be potentially insufficient for AC lesions.

### Supplementary Information

Below is the link to the electronic supplementary material.

Supplementary file1 (DOCX 16 KB)
