# Supplementary material for: Low-Dose Radiation Yields Lower Rates of Pathologic Response in Esophageal Cancer Patients
Source: Ann Surg Oncol. 2024 Jan 10;31(4):2499–508. doi: 10.1245/s10434-023-14810-8 (PMC10908612; doi:10.1245/s10434-023-14810-8)
Supplement: Supplementary file 1 — Supplementary file1 (DOCX 16 KB) [file 10434_2023_14810_MOESM1_ESM.docx]

Supplemental material 1. Cox regression analysis for overall survival

|  | Unadjusted HR | 95%CI | P-value | Adjusted HR | 95%CI | P-value |
| --- | --- | --- | --- | --- | --- | --- |
| cT stage  1  2  3  4 | 1  1.62  2.64  2.52 | 0.38-6.91  0.65-10.67  0.42-15.12 | 0.094 | 1.32  2.01  1.53 | 0.30-5.73  0.49-8.32  0.25-9.47 | 0.708  0.333  0.644 |
| cN stage  0  1  2  3 | 1  0.98  0.15  1.56 | 0.71-1.35  0.02-1.08  0.38-6.41 | 0.157 |  |  |  |
| pCR | 0.59 | 0.42-0.85 | 0.003 | 0.58 | 0.21-1.58 | 0.287 |
| pT stage  0  1  2  3  4 | 1  1.14  1.42  1.85  4.07 | 0.67-1.96  0.88-2.31  1.28-2.66  1.60-10.34 | 0.621  0.153  <0.001  0.003 | 1  0.65  0.70  0.78  1.60 | 0.23-1.79  0.25-1.98  0.29-2.11  0.41-6.27 | 0.403  0.503  0.629  0.498 |
| pN stage  0  1  2  3 | 1  1.19  2.38  2.59 | 0.84-1.69  1.56-3.64  1.30-5.14 | 0.314  <0.001  0.007 | 1  0.97  1.98  1.77 | 0.66-1.43  1.25-3.13  0.83-3.76 | 0.903  0.003  0.137 |
| Major complications | 1.99 | 1.47-2.72 | <0.001 | 2.18 | 1.58-3.01 | <0.001 |
| R1/2 resection | 3.29 | 2.05-5.28 | <0.001 | 2.26 | 1.35-2.76 | 0.002 |
| Radiation group  41.4Gy  45 Gy  50.4 Gy | 1  1.04  0.97 | 0.66-1.67  0.59-1.58 | 0.893 |  |  |  |
| Chemotherapy  5FU-Cisplatin  FOLFOX  Carbo-taxol  Other | 1  0.78  0.72  1.23 | 0.54-1.12  0.47-1.08  0.87-1.62 | 0.457 |  |  |  |
| Histology  SCC  AC | 1  0.89 | 0.66-1.19 | 0.434 |  |  |  |

HR= Hazard Ratio, 95%CI= 95% Confidence Intervals, pCR= pathologic complete response, SCC= Squamous Cell Cancer ; AC= adenocarcinoma
